# The acute and long-term management of food allergy: protocol for a rapid systematic review

**DOI:** 10.1186/2045-7022-3-12

**Published:** 2013-03-30

**Authors:** Debra de Silva, Sukhmeet S Panesar, Sundeep Thusu, Tamara Rader, Thomas Werfel, Antonella Muraro, Karin Hoffmann-Sommergruber, Graham Roberts, Aziz Sheikh

**Affiliations:** 1 The Evidence Centre, 126 Central Avenue, London TW3 2RJ, UK; 2 University of Edinburgh, Teviot Place, Edinburgh EH8 9AG, UK; 3 Centre for Transcultural Oral Health, King’s College London, Strand, London WC2R 2LS, UK; 4 University of Ottawa, 75 Laurier Avenue East, Ottawa, ON K1N 6N5, Canada; 5 Hanover Medical School, Carl-Neuberg-Straße 1, Hanover 30625, Germany; 6 Padua General University Hospital, Via Giustiniani 3, Padua 35128, Italy; 7 Medical University of Vienna, Spitalgasse 23, Vienna 1090, Austria; 8 Faculty of Medicine, University of Southampton, Southampton, SO171BJ, UK

**Keywords:** Food allergy, LgE-mediated, Management, Treatment

## Abstract

**Background:**

Allergic reactions to plant and animal derived food allergens can have serious consequences for sufferers and their families. The associated social, emotional and financial costs make it a priority to understand the best ways of managing such immune-mediated hypersensitivity responses. Conceptually, there are two main approaches to managing food allergy: those targeting immediate symptoms and those aiming to support long-term management of the condition. The European Academy of Allergy and Clinical Immunology is developing guidelines about what constitutes an effective treatment for food allergies. As part of the guidelines development process, a systematic review is planned to examine published research about the management of food allergy in adults and children.

**Methods:**

Seven bibliographic databases were searched from their inception to September 30, 2012 for systematic reviews, randomized controlled trials, quasi-randomized controlled trials, controlled clinical trials, controlled before-and-after studies and interrupted time series. Experts were consulted for additional studies. There were no language or geographic restrictions. Studies were critically appraised using the Critical Appraisal Skills Program and Cochrane EPOC Risk of Bias tools. Only studies where people had a diagnosis of food allergy or reported a history of food allergy were included. This means that many studies of conditions that may be caused by food allergy are omitted, because only research in people with an explicit diagnosis or history was eligible.

**Discussion:**

Many initiatives have been tested to treat the immediate symptoms of food allergy (acute management) and to deal with longer lasting symptoms or induce tolerability to potential allergens (long-term management). The best management strategies for people with food allergy are likely to depend on the type of allergy, symptom manifestations and age. There is a real need to increase the amount of high quality research devoted to treatment strategies for food allergy. Food allergy can be debilitating and is affecting an increasing number of children and adults. With such little known about how to effectively manage the condition and its manifestations, this appears a priority for future research.

## Background

Recent estimates suggest that around 17 million people in Europe suffer from allergies triggered by foods such as milk, eggs, peanuts, tree nuts or seafood, and an increasing number are seeking treatment through primary care and hospital emergency departments [1]. Food allergies can have a significant effect on people’s quality of life and physical functioning and can also be costly in terms of medical visits and treatments [1]. With such high social and economic costs, it is important to ensure that effective, evidence-based treatments are available.

The European Academy of Allergy and Clinical Immunology (EAACI) is developing guidelines about what constitutes an effective treatment. As part of the guidelines development process, a systematic review is planned to examine published research about the management of food allergy in adults and children.

### Aims

The aims of this systematic review will be to:

•examine what pharmacological and non-pharmacological interventions have been researched to manage the symptoms of food allergy in individuals (i.e. acute treatment)

•examine what pharmacological and non-pharmacological interventions have been researched to manage longer-term outcomes in individuals such as tolerance and coping (i.e. longer-term management)

•where possible, quantify the extent to which such approaches may be effective.

### Scope

The umbrella term ‘food hypersensitivity’ is used to describe any adverse reaction to food [2]. The term ‘food allergy’ refers to the subgroup of food-triggered reactions in which immunological mechanisms have been implicated, whether IgE-mediated, non-IgE-mediated, or involving a combination of IgE- and non-IgE-mediated etiologies [3]. All other reactions to food that have sometimes been referred to as ‘food intolerance’ constitute non-allergic food hypersensitivity reactions and are outside the focus of this enquiry.

The topic of food allergy is complicated, however, by the fact that IgE-mediated reactions can manifest as angioedema, urticaria, atopic eczema/dermatitis, oral allergy syndrome and anaphylaxis, for example. Non-IgE-mediated immunological reactions result from activation of other immunological pathways (e.g. T-cell mediated) and can manifest as atopic eczema/dermatitis, gastro-esophageal reflux disease, food protein-induced enterocolitis, proctocolitis and enteropathy syndromes. The contemporary definition of food allergy thus includes several clinical entities with different pathophysiologies resulting from exposure to different foods [4]. Table 1 lists the potential manifestations of food allergy included in the review.

**Table 1:** Potential manifestations of food allergy eligible for inclusion

***Pathology***	***Disorder***
IgE-mediated (acute-onset)	Acute urticaria/angioedema
	Contact urticaria
	Oral allergy syndrome (pollen-associated food allergy syndrome)
	Allergic asthma/wheeze
	Atopic eczema/dermatitis
	Immediate gastrointestinal hypersensitivity such as vomiting and diarrhoea
Cell-mediated (delayed onset/chronic)	Food protein induced gastroenteropathy
	Food protein-induced enterocolitis syndrome
	Food protein-induced allergic proctocolitis
	Atopic eczema/dermatitis
	Allergic contact dermatitis
	Heiner syndrome
Combined IgE and cell-mediated (delayed onset/chronic)	Atopic eczema/dermatitis
	Eosinophilic esophagitis
	Eosinophilic gastroenteritis

Food allergy is complex and may manifest as other pathologies. As well as food allergy *per se*, studies of the following pathologies are eligible for inclusion, as long as there is suspicion of food allergy and the intervention focuses on addressing dietary or food-related issues. Coeliac disease is an important non-IgE mediated condition but as it has distinct symptoms and prognosis different from atopic conditions it will be excluded from this review.

The clinical management of food allergy includes strategies to minimise the risk of further reactions, primarily through education and behavioural approaches to avoid allergens and dietary modification, and approaches to improve outcomes if a further reaction does occur through pharmacological and non-pharmacological management strategies. There is also growing interest in the effectiveness of potential immuno-modulatory treatment approaches, including sublingual and oral immunotherapy [2]. The review will synthesise evidence about the effectiveness of these management approaches for individuals. Interventions at a community, organisational or societal level, such as food labeling or regulation, will not be included.

The EAACI is in the process of developing the *EAACI Guideline for Food Allergy and Anaphylaxis*, and this systematic review is one of seven inter-linked evidence syntheses that are being undertaken in order to provide a state-of-the-art synopsis of the current evidence base in relation to epidemiology, prevention, diagnosis and clinical management, and impact on quality of life, which will be used to inform clinical recommendations.

## Methods

### Inclusion criteria

**Figure 1:**
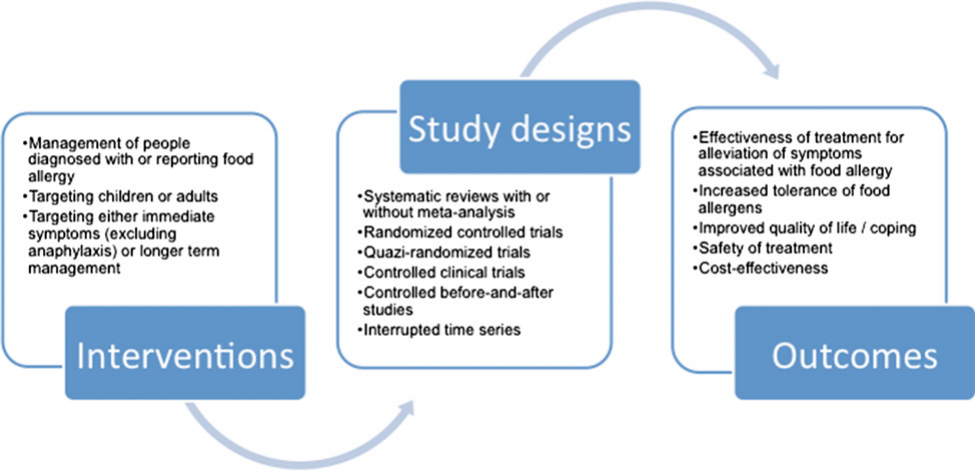
Conceptualisation of systematic review on the management of food allergy.

We have conceptualised the review to incorporate the interventions, study designs and outcomes, as shown in Figure 1: Conceptualisation of systematic review on the management of food allergy.

Study designs eligible for inclusion in the review comprise:

•systematic reviews with or without meta-analyses

•randomised controlled trials

•quazi-randomised controlled trials and controlled clinical trials (defined as studies where the comparison group is not fully randomised)

•controlled before and after studies (only where a clearly defined comparison group is available prospectively) and interrupted time series studies (where measures are taken during at least three timepoints before and at least three time points after intervention).

We suspect that there will be limited information available from systematic reviews and randomised trials, so we have opted to incude lower forms of evidence where non-random allocation of patients has occurred [5]. Studies already included in other systematic reviews will also be eligible for quality appraisal and inclusion in this review. Only research published as full papers will be eligible for inclusion. Where repeated reports of the same study are identified, the most up-to-date or detailed will be included.

### Exclusion criteria

The following material will be excluded from the review:

•non-systematic reviews, discussion papers, non-research letters and editorials

•qualitative studies

•case studies, case series, non controlled before and after studies and other lower quality designs

•animal studies

•abstracts and studies not available in full form

•unpublished material

•studies about anaphylaxis (as these are covered in another review in the series).

### Search strategy

We will search the following databases:

•Cochrane Library, including:

○ Cochrane Database of Systematic Reviews (CDSR)

○ Database of Reviews of Effectiveness (DARE)

○ CENTRAL (Trials)

○ Methods Studies

○ Health Technology Assessments (HTA)

○ Economic Evaluations Database (EED)

•MEDLINE (OVID)

•Embase(OVID)

•CINAHL (Ebscohost)

•ISI Web of Science (Thomson Web of Knowledge)

•TRIP Database (http://www.tripdatabase.com)

•Clinicaltrials.gov (NIH web)

A highly sensitive search strategy has been developed, and validated study design filters will be applied to retrieve all articles pertaining to the management of food allergy from electronic bibliographic databases.

To retrieve systematic reviews, we will use the systematic review filter developed at McMaster University Health Information Research Unit (HIRU) [6].

To retrieve randomised controlled trials, we will apply the Cochrane highly sensitive search strategy for identifying randomised trials in MEDLINE: sensitivity- and precision-maximising version (2008 revision); Ovid format from Chapter 6 of the Cochrane Handbook [7]. To retrieve non-randomised studies such as interrupted time-series (ITS), controlled before-and-after (CBA) studies and controlled clinical trials (CCT), we will use the Cochrane Effective Practice and Organisation of Care (EPOC) filter Version 2.4 from the EPOC Group [8,9].

The search strategy has been developed on OVID MEDLINE and then adapted for the other databases (see Additional file 1 for full search strategies). In all cases the databases will be searched from inception to 30 September 2012. All references will be imported into an EndNote Library and tagged with the name of the database. Additional references will be located through searching the references cited in identified reviews and contacting experts in the field.

We will invite experts who are active in the field from a range of disciplines and geography to comment on our search strategy, and the list of included studies. No language restrictions will be applied and, where possible, all literature will be translated.

### Study selection

Identified titles will be checked independently by two reviewers according to the above selection criteria and categorised as: included, not included and unsure. For those papers in the unsure category, we will retrieve the abstract and re-categorise as above. Any discrepancies will be resolved by consensus and if necessary a third reviewer will be consulted. Full text copies of potentially relevant studies will be obtained and their eligibility for inclusion independently assessed. Studies that do not fulfil all of the inclusion criteria will be excluded.

### Quality assessment strategy

Quality assessments will independently be carried out on each study by two reviewers. Systematic reviews will be assessed for quality using the relevant Critical Appraisal Skills Programme Tool (CASP) [10]. We will assess the risk of bias of studies eligible for the review using the criteria suggested by EPOC [11]. Randomised controlled trials, controlled clinical trials and controlled before and after studies will be assessed for generation of allocation sequence, concealment of allocation, baseline outcome measurements, baseline characteristics, incomplete outcome data, blinding of outcome assessor, protection against contamination, selective outcome reporting and other risks of bias. For interrupted time series designs we will also assess the independence of the intervention from other changes, the pre-specified shape of the intervention and whether the intervention was likely to affect data collection. Our quality assessments will draw on the principles incorporated into the Cochrane EPOC Group guidelines for assessing intervention studies [12] and the Strengthening the Reporting of Observational Studies in Epidemiology (STROBE) for assessing observational studies [13].

### Analysis, data synthesis and reporting

Data will be independently extracted onto a customised data extraction sheet by two reviewers, and any discrepancies will be resolved by discussion or, if agreement cannot be reached, by arbitration by a third reviewer.

A descriptive summary with data tables will be produced to summarise the literature. A narrative synthesis of the data will be undertaken. If clinically and statistically appropriate, meta-analysis using either fixed-effect or random-effects modelling may be undertaken for potentially useful interventions using methods suggested by Agresti and Coul [14].

This review has been registered with the International Prospective Register of Systematic Reviews (PROSPERO) and has registration number CRD42013003708 allocated to it. The Preferred Reporting Items for Systematic Reviews and Meta-Analyses (PRISMA) checklist will be used to guide the reporting of the systematic review [15].

## Discussion

Many initiatives have been tested to treat the immediate symptoms of food allergy (acute management) and to deal with longer lasting symptoms or induce tolerability to potential allergens (long-term management). The best management strategies for people with food allergy are likely to depend on the type of allergy, symptom manifestations and age. In terms of acute management, there is little evidence to help differentiate the most effective treatments. Regarding long-term management, avoiding the culprit food or substituting an alternative is likely to be of benefit. Other management strategies include alternatives to cow’s milk formula to reduce symptoms in infants with cow’s milk allergy, supplements such as probiotics and allergen-specific immunotherapy. Most importantly, this review will help to synthesis the highest levels of evidence pertaining to treatment strategies for food allergy and identify any gaps in our knowledge. Food allergy can be debilitating and is affecting an increasing number of children and adults. With such little known about how to effectively manage the condition and its manifestations, this appears a priority for future research.

## Abbreviations

CASP: Critical appraisal skills programme tool; CBA: Controlled before-and-after studies; CDSR: Cochrane database of systematic reviews; DARE: Database of reviews of effectiveness; EAACI: European academy of allergy and clinical immunology; EED: Economic evaluations database; EPOC: Effective practice and organisation of care; HTA: Health technology assessments; PROSPERO: Prospective register of systematic reviews; PRISMA: Preferred reporting items for systematic reviews and meta-analyses; STROBE: Strengthening the reporting of observational studies in epidemiology.

## Competing interests

The authors declare that they have no competing interests, financial or otherwise.

## Authors’ contributions

DdeS, SSP, ST and TR conceptualised and designed the protocol and drafted earlier versions of the document in their capacity as methodologists. TW, AM, KH-S and GR contributed to further refinements of the protocol and revised it critically for important intellectual content in their capacity as guideline leads. AS led on the development of concepts used in this protocol and revised it critically for important intellectual content in his capacity as the methodology lead. All authors approved the final version to be published.

## Supplementary Material

Additional file 1 Search strategies. Click here for file
