# Supplementary material for: The acute and long-term management of food allergy: protocol for a rapid systematic review
Source: Clin Transl Allergy. 2013 Mar 30;3:12. doi: 10.1186/2045-7022-3-12 (PMC3637062; doi:10.1186/2045-7022-3-12)
Supplement: Additional file 1 — Search strategies. [file 2045-7022-3-12-S1.docx]

**Appendix 1: Search strategies**

*Database: Ovid MEDLINE(R) In-Process & Other Non-Indexed Citations and Ovid MEDLINE(R) <1946 to Present>*

Search Strategy:

----------------------------------------------------------------------------

| 1 | exp Food Hypersensitivity/ |
| --- | --- |
| 2 | food allerg*.mp. |
| 3 | food hypersensitivity.mp. |
| 4 | food hypersensitivities.mp. |
| 5 | allergy, food.mp. |
| 6 | or/1-5 |
| 7 | (rat or rats or cow or cows or chicken? or horse or horses or mice or mouse or bovine or animal?).ti. |
| 8 | exp animals/ not humans.sh. |
| 9 | 7 or 8 |
| 10 | 6 not 9 |
| 11 | MEDLINE.tw. |
| 12 | systematic review.tw. |
| 13 | meta analysis.pt. |
| 14 | or/11-13 |
| 15 | randomized controlled trial.pt. |
| 16 | controlled clinical trial.pt. |
| 17 | randomized.ab. |
| 18 | placebo.ab. |
| 19 | clinical trials as topic.sh. |
| 20 | randomly.ab. |
| 21 | trial.ti. |
| 22 | or/15-21 |
| 23 | intervention?.ti. or (intervention? adj6 (clinician? or collaborat$ or community or complex or DESIGN$ or doctor? or educational or family doctor? or family physician? or family practitioner? or financial or GP or general practice? or hospital? or impact? or improv$ or individuali?e? or individuali?ing or interdisciplin$ or multicomponent or multi-component or multidisciplin$ or multi-disciplin$ or multifacet$ or multi-facet$ or multimodal$ or multi-modal$ or personali?e? or personali?ing or pharmacies or pharmacist? or pharmacy or physician? or practitioner? or prescrib$ or prescription? or primary care or professional$ or provider? or regulatory or regulatory or tailor$ or target$ or team$ or usual care)).ab. |
| 24 | (pre-intervention? or preintervention? or "pre intervention?" or post-intervention? or postintervention? or "post intervention?").ti,ab. |
| 25 | (hospital$ or patient?).hw. and (study or studies or care or health$ or practitioner? or provider? or physician? or nurse? or nursing or doctor?).ti,hw. |
| 26 | demonstration project?.ti,ab. |
| 27 | (pre-post or "pre test$" or pretest$ or posttest$ or "post test$" or (pre adj5 post)).ti,ab. |
| 28 | (pre-workshop or post-workshop or (before adj3 workshop) or (after adj3 workshop)).ti,ab. |
| 29 | trial.ti. or ((study adj3 aim?) or "our study").ab. |
| 30 | (before adj10 (after or during)).ti,ab. |
| 31 | ("quasi-experiment$" or quasiexperiment$ or "quasi random$" or quasirandom$ or "quasi control$" or quasicontrol$ or ((quasi$ or experimental) adj3 (method$ or study or trial or design$))).ti,ab,hw. |
| 32 | ("time series" adj2 interrupt$).ti,ab,hw. |
| 33 | (time points adj3 (over or multiple or three or four or five or six or seven or eight or nine or ten or eleven or twelve or month$ or hour? or day? or "more than")).ab. |
| 34 | pilot.ti. |
| 35 | Pilot projects/ |
| 36 | (clinical trial or controlled clinical trial or multicenter study).pt. (589305) |
| 37 | (multicentre or multicenter or multi-centre or multi-center).ti. (24808) |
| 38 | random$.ti,ab. or controlled.ti. |
| 39 | (control adj3 (area or cohort? or compare? or condition or design or group? or intervention? or participant? or study)).ab. not (controlled clinical trial or randomized controlled trial).pt. |
| 40 | comment on.cm. or review.ti,pt. or randomized controlled trial.pt. |
| 41 | or/23-40 |
| 42 | 10 and 14 |
| 43 | 10 and 22 |
| 44 | 10 and 41 |
| 45 | or/42-44 |

*Database: Embase Classic+Embase <1990 to 2012 August 20>*

Search Strategy:

--------------------------------------------------------------------------------

*Database: Embase Classic+Embase <1947 to 2012 September 17>*

Search Strategy:

--------------------------------------------------------------------------------

| 1 | exp Food Hypersensitivity/ |
| --- | --- |
| 2 | food allerg*.mp. |
| 3 | food hypersensitivity.mp. |
| 4 | food hypersensitivities.mp. |
| 5 | allergy, food.mp. |
| 6 | or/1-5 |
| 7 | (rat or rats or cow or cows or chicken? or horse or horses or mice or mouse or bovine or animal?).ti. |
| 8 | (animal$ not human$).sh,hw. |
| 9 | 7 or 8 |
| 10 | 6 not 9 |
| 11 | intervention?.ti. or (intervention? adj6 (clinician? or collaborat$ or community or complex or DESIGN$ or doctor? or educational or family doctor? or family physician? or family practitioner? or financial or GP or general practice? or hospital? or impact? or improv$ or individuali?e? or individuali?ing or interdisciplin$ or multicomponent or multi-component or multidisciplin$ or multi-disciplin$ or multifacet$ or multi-facet$ or multimodal$ or multi-modal$ or personali?e? or personali?ing or pharmacies or pharmacist? or pharmacy or physician? or practitioner? or prescrib$ or prescription? or primary care or professional$ or provider? or regulatory or regulatory or tailor$ or target$ or team$ or usual care)).ab. (172132) |
| 12 | (pre-intervention? or preintervention? or "pre intervention?" or post-intervention? or postintervention? or "post intervention?").ti,ab. [added 2.4] (10005) |
| 13 | [or/11-36] |
| 14 | [or/38-40] |
| 15 | [or/42-56] |
| 16 | [or/61-63] |
| 17 | *food allergy/ |
| 18 | *nutritional intolerance/ |
| 19 | *Food Hypersensitivity/ |
| 20 | ((food$ or nutrient$) adj5 (allerg$ or hypersensitiv$ or sensitiv$ or intoleran$ or reaction$)).ti,ab. |
| 21 | oral allerg$.ti,ab. |
| 22 | or/17-21 |
| 23 | intervention?.ti. or (intervention? adj6 (clinician? or collaborat$ or community or complex or DESIGN$ or doctor? or educational or family doctor? or family physician? or family practitioner? or financial or GP or general practice? or hospital? or impact? or improv$ or individuali?e? or individuali?ing or interdisciplin$ or multicomponent or multi-component or multidisciplin$ or multi-disciplin$ or multifacet$ or multi-facet$ or multimodal$ or multi-modal$ or personali?e? or personali?ing or pharmacies or pharmacist? or pharmacy or physician? or practitioner? or prescrib$ or prescription? or primary care or professional$ or provider? or regulatory or regulatory or tailor$ or target$ or team$ or usual care)).ab. |
| 24 | (pre-intervention? or preintervention? or "pre intervention?" or post-intervention? or postintervention? or "post intervention?").ti,ab. [added 2.4] (10005) |
| 25 | (hospital$ or patient?).hw. and (study or studies or care or health$ or practitioner? or provider? or physician? or nurse? or nursing or doctor?).ti,hw. (1422994) |
| 26 | demonstration project?.ti,ab. |
| 27 | (pre-post or "pre test$" or pretest$ or posttest$ or "post test$" or (pre adj5 post)).ti,ab. (78798) |
| 28 | (pre-workshop or post-workshop or (before adj3 workshop) or (after adj3 workshop)).ti,ab. (665) |
| 29 | trial.ti. or ((study adj3 aim?) or "our study").ab. |
| 30 | (before adj10 (after or during)).ti,ab. |
| 31 | (time points adj3 (over or multiple or three or four or five or six or seven or eight or nine or ten or eleven or twelve or month$ or hour? or day? or "more than")).ab. |
| 32 | pilot.ti. |
| 33 | intervention?.ti. or (intervention? adj6 (clinician? or collaborat$ or community or complex or DESIGN$ or doctor? or educational or family doctor? or family physician? or family practitioner? or financial or GP or general practice? or hospital? or impact? or improv$ or individuali?e? or individuali?ing or interdisciplin$ or multicomponent or multi-component or multidisciplin$ or multi-disciplin$ or multifacet$ or multi-facet$ or multimodal$ or multi-modal$ or personali?e? or personali?ing or pharmacies or pharmacist? or pharmacy or physician? or practitioner? or prescrib$ or prescription? or primary care or professional$ or provider? or regulatory or regulatory or tailor$ or target$ or team$ or usual care)).ab. |
| 34 | (pre-intervention? or preintervention? or "pre intervention?" or post-intervention? or postintervention? or "post intervention?").ti,ab. [added 2.4] |
| 35 | (hospital$ or patient?).hw. and (study or studies or care or health$ or practitioner? or provider? or physician? or nurse? or nursing or doctor?).ti,hw. |
| 36 | demonstration project?.ti,ab. |
| 37 | (pre-post or "pre test$" or pretest$ or posttest$ or "post test$" or (pre adj5 post)).ti,ab. |
| 38 | (pre-workshop or post-workshop or (before adj3 workshop) or (after adj3 workshop)).ti,ab. |
| 39 | trial.ti. or ((study adj3 aim?) or "our study").ab. |
| 40 | (before adj10 (after or during)).ti,ab. |
| 41 | (time points adj3 (over or multiple or three or four or five or six or seven or eight or nine or ten or eleven or twelve or month$ or hour? or day? or "more than")).ab. |
| 42 | pilot.ti. |
| 43 | (multicentre or multicenter or multi-centre or multi-center).ti. |
| 44 | random$.ti,ab. or controlled.ti. |
| 45 | review.ti. [EM] |
| 46 | *experimental design/ or *pilot study/ or quasi experimental study/ [EM] (5126) |
| 47 | ("quasi-experiment$" or quasiexperiment$ or "quasi random$" or quasirandom$ or "quasi control$" or quasicontrol$ or ((quasi$ or experimental) adj3 (method$ or study or trial or design$))).ti,ab. [EM] (118829) |
| 48 | ("time series" adj2 interrupt$).ti,ab. [EM] |
| 49 | or/23-48 |
| 50 | meta-analys:.mp. |
| 51 | search:.tw. |
| 52 | review.pt. |
| 53 | or/50-52 |
| 54 | random$.tw. |
| 55 | factorial$.tw. |
| 56 | crossover$.tw. |
| 57 | cross over.tw. |
| 58 | cross-over.tw. |
| 59 | placebo$.tw. |
| 60 | (doubl$ adj blind$).tw. |
| 61 | (singl$ adj blind$).tw. |
| 62 | assign$.tw. |
| 63 | allocat$.tw. |
| 64 | volunteer$.tw. |
| 65 | crossover procedure/ |
| 66 | double blind procedure/ |
| 67 | randomized controlled trial/ |
| 68 | single blind procedure/ |
| 69 | or/54-68 |
| 70 | 22 and 49 |
| 71 | 22 and 53 |
| 72 | 22 and 69 |
| 73 | or/70-72 |
| 74 | 73 not 9 |

*Database: CINAHLSearch Strategy:*

| S9 | S1 or S8 |
| --- | --- |
| S8 | S6 and S7 |
| S7 | S4 or S5 |
| S6 | S2 or S3 |
| S5 | AB allergy or allergic or hypersensitive or hypersensitivity or sensitive or sensitivity or intolerant or intolerance or reaction |
| S4 | TI allergy or allergic or hypersensitive or hypersensitivity or sensitive or sensitivity or intolerant or intolerance or reaction |
| S3 | AB food or nutrient |
| S2 | TI food or nutrient |
| S1 | (MM "Food Hypersensitivity") |

*Database: ISI Web of Science: Science Citation Index, Conference Proceedings*

*Citation*

Search strategy:

# 2

Topic=(food or nutrient) AND Topic=(allergy or allergic or hypersensitive or hypersensitivity or sensitive or sensitivity or intolerant or intolerance or reaction )

Refined by: Web of Science Categories=( ALLERGY OR IMMUNOLOGY ) AND Document Types=( PROCEEDINGS PAPER OR MEETING ABSTRACT )

Databases=CPCI-S Timespan=All Years

Lemmatization=On

# 1

Topic=(food or nutrient) AND Topic=(allergy or allergic or hypersensitive or hypersensitivity or sensitive or sensitivity or intolerant or intolerance or reaction )

Databases=CPCI-S Timespan=All Years

Lemmatization=On

*Database: Cochrane Library*

Search strategy:

#1 MeSH descriptor Food Hypersensitivity explode all trees

#2 (food hypersensitivity or (food* and (allergy or allergies or allergic or allergen*)))

#4 (#1 OR #2)

*Database: TRIP Database*

Search Strategy: (Advanced search screen)

area:"Allergies and Immunology"

any of these words: food allerg*

Downloaded: Evidence Based Synopses, Systematic Reviews, Guidelines

All years

*Database: Clinicaltrials.gov*

Search Strategy: (Advanced search screen)

Conditions: food allergy or food intoleran* or food reaction or food hypersensitiv* or food sensitiv*

all years
